# From textile engineering to bioelectronics: an interview with Sahika Inal on multidisciplinarity and the exciting field of biosensors

**DOI:** 10.1038/s42003-021-02654-5

**Published:** 2021-09-20

## Abstract

Sahika Inal is an Associate Professor of Bioengineering at KAUST and has been leading the Organic Bioelectronics group since 2016. With a Ph.D. in Experimental Physics from the University of Potsdam (Potsdam, Germany) and a postdoctoral fellowship in the Department of Bioelectronics at the Centre Microelectronique de Provence in France, she is an expert in the characterization of conjugated polymers and biomedical device development. In this Q&A, Dr. Inal tells us about her research interests, excitement of constantly learning in the lab and the expanding biosensor field.

**Figure:**
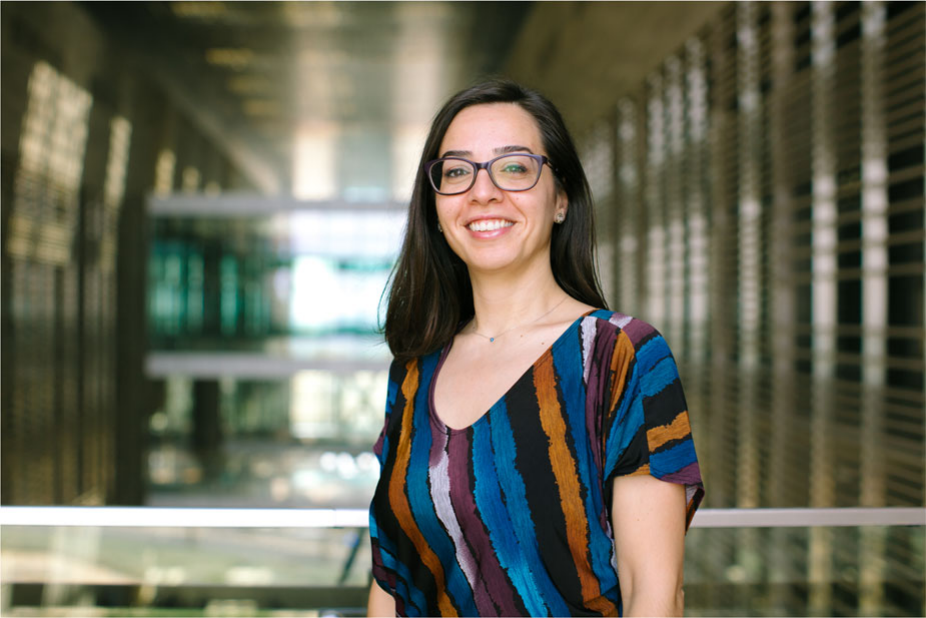
Anastasia Serin

Please tell us about your research interests.

My research focuses on bioelectronic materials and devices. I like playing with functional polymers and making them more functional than they are meant to be! We then leverage these functionalities when interfacing with biological systems with the devices we build.

With a background in textile engineering, how did you embark on this journey towards addressing health issues with bioelectronics?

I have always been interested in “smart” materials. Although textile engineering may sound far off, the polymers used in textiles have some special powers and importantly, they interface with the human skin. Having learnt engineering principles, how to handle polymers, and transform them into useful products for end users, I have acquired the right background to design polymer-based devices for healthcare. The crucial point in my career has been the MSc studies I did after my engineering degree as I learnt more about the fundamentals of polymers, and where I discovered the “electronic” ones. After working with polymers that have distinct optical and electronic properties for pathogen detection in my Ph.D., I knew that I needed to learn more biology and get hands-on experience. I was lucky enough to find a postdoc in a team that offered training next to biologists and clinicians. The work that I did in that interdisciplinary environment opened up my horizons. The ideas that I pursue in my lab today appeared back then as I was interacting with biologists, working with them at the wet bench while being advised by a biochemist. Overall, the diversity in my training is what I tap into when designing devices for diagnostics and therapy.

Biology, electronics and material are such diverse fields that you explore. What are the challenges of being multidisciplinary?

The language used by these different disciplines may sound foreign to one another in the beginning. Communicating using the right vocabulary is a challenge that I see in our lab when people with different backgrounds are put in a room for the first time to work on a project together. It takes patience to understand each other. Those that are open to learning new languages and welcoming novel ideas overcome this initial blurry phase quickly. I guess being multidisciplinary is like living in a foreign country without being very proficient with the language. It is exciting but also takes a lot of attention to detail to set everything right as one may miss important points due to the loss in translation.

What general advice would you give on how to establish a successful academic career?

I am not sure if I am senior enough to give advice on being successful! But to be happy in academia, one needs to make sure that she is excited about research. There may be things that upset a person in an academic environment, but I know that I am lucky to be given tools to play with. Every day in the lab is like the playground to discover and learn something new.

Tell us about the emerging research environment in Saudi Arabia?

Saudi Arabia is heavily invested in research and innovation, and makes all the necessary efforts to become competitive. Besides KAUST, there are other universities and institutions that perform research with topics that are critical to the wellbeing of our world, such as clean energy and climate change.

Biosensors constitute a very exciting research field with fascinating possibilities. Which development are you most excited about?

The pandemic showed a gap in our diagnostic toolbox and the immediate need for affordable, portable and rapid tools that can detect the virus. As such, research from several teams (including ours) have started to focus more on translational activities. I find it very exciting that, globally, there is more funding to support such efforts and bring lab-based technologies that have the potential to change the pace of diagnostics into maturation.

To learn more about Dr. Inal’s research, visit her lab website (https://bioel.kaust.edu.sa/) or follow her on twitter @InalSahika.

*This interview was conducted by Associate Editor Anam Akhtar*.

